# Beyond Occam's Razor: Concurrent Non-functional Pituitary Macroadenoma and Metastatic Hepatocellular Carcinoma in a Patient With Functional Cure of Hepatitis B

**DOI:** 10.7759/cureus.62339

**Published:** 2024-06-13

**Authors:** Yu-Ming Li, Ching-Shiang Shaw, Tse-Ching Chen, Diego Shih-Chieh Lin

**Affiliations:** 1 Department of Integrative Immunobiology, Duke University School of Medicine, Durham, USA; 2 Department of Medicine, Koo Foundation Sun Yat-Sen Cancer Center, Taipei, TWN; 3 Department of Pathology, Chang Gung Memorial Hospital, Taoyuan, TWN; 4 Department of Pathology and Laboratory Medicine, Taipei Veterans General Hospital, Taipei, TWN

**Keywords:** panhypopituitarism, euvolemic hypotonic hyponatremia, hickam’s dictum, occam's razor, functional cure, hepatitis b virus, hepatocellular carcinoma (hcc), nonfunctioning pituitary adenoma, chronic fatigue

## Abstract

Occam’s razor, the principle of parsimony, is frequently employed in medicine to derive a single diagnosis from a patient’s myriad symptoms. Conversely, Hickam’s dictum, which embraces the principle of plenitude by considering multiple diagnoses for a patient’s presentation, is often underutilized or not as widely recognized as Occam’s razor. The application of Hickam's dictum is particularly crucial when evaluating nonspecific symptoms such as fatigue, which can manifest in various diseases. This report describes the case of a 72-year-old man with a history of functional cure for hepatitis B who presented with chronic fatigue and hyponatremia. Initially, he was diagnosed with non-functional pituitary macroadenoma and panhypopituitarism. Two months following pituitary surgery, the onset of dyspepsia and the recurrence of fatigue revealed metastatic tumors in the liver, stomach, pancreas, left adrenal gland, and peri-pancreatic lymph nodes. A liver biopsy confirmed the diagnosis of hepatocellular carcinoma. This case highlights the importance of considering multiple, potentially co-existing conditions based on the patient’s symptoms and risk factors to complete the thorough diagnoses. Additionally, it emphasizes the need to remain vigilant regarding the risk of liver cancer in patients with a history of chronic hepatitis B infection, irrespective of a functional cure.

## Introduction

Fatigue is a common chief complaint in primary care, representing about 5-10% of consultations, particularly among the elderly [1]. Despite its prevalence, at least one-third of these patients may not receive a definite diagnosis due to its vague and non-specific characteristics [2]. Occasionally, fatigue might be the first indication of serious underlying conditions, emphasizing the necessity for a comprehensive evaluation and extensive differential diagnosis for proper identification. Given the variety of possible underlying conditions, fatigue poses a complex clinical challenge that requires attention not only just as a symptom of a specific disease but as a manifestation of multiple health issues.

In the context of non-functional pituitary adenoma (NFPA), fatigue can result from secondary adrenal insufficiency or hypothyroidism and is often accompanied by symptoms like poor appetite, weight loss, and a depressed mood. These subtle symptoms can easily be misattributed to normal aging, leading to a delay in the diagnosis of NFPA by two to five years [3]. Frequently, NFPA is diagnosed only when the adenoma grows large enough to cause a mass effect, significantly compressing the optic nerve and resulting in visual defects. Therefore, recognition of additional signs suggestive of NFPA is essential for guiding an early diagnosis. Notably, hyponatremia is a common electrolyte imbalance that can be found in about 10% of patients with NFPA [4]. This type of hyponatremia is hypotonic and arises from the action of antidiuretic hormone (ADH) on the collecting tubules in the kidneys, leading to free water reabsorption. Unlike the typical cases where hypovolemia triggers ADH secretion, patients with NFPA who present with hyponatremia are usually euvolemic. Despite having adequate intravascular volume, the loss of inhibition from cortisol or thyroid hormone results in inappropriate ADH secretion [5]. Although hyponatremia has significant diagnostic value, the link between hyponatremia and NFPA with hypopituitarism often remains unrecognized [6]. This underscores the need for thorough evaluations, particularly in elderly patients presenting with fatigue and hyponatremia.

Fatigue is also prevalent among patients with malignancies like hepatocellular carcinoma (HCC), where it may be the only early symptom. This subtle presentation can lead to delayed diagnosis and negatively impact patient outcomes. Hepatitis B virus (HBV) is one of the main risk factors for HCC development. Current guidelines recommend regular HCC surveillance in patients with chronic hepatitis B, especially those with HBV infection-induced liver cirrhosis [7,8]. Achieving a functional cure of hepatitis B, characterized by undetectable serum HBV surface antigen (HBsAg) and HBV DNA, significantly reduces the risk of HCC [9]. However, emerging evidence indicates that HCC can still develop in non-cirrhotic patients who have achieved a functional cure [10,11]. Furthermore, factors such as male gender and age over 50 have been identified as significant risk factors for HCC among patients with HBsAg seroclearance [10,11]. Despite current guidelines starting to recommend continuous surveillance for HCC in patients with a functional cure of hepatitis B, there might be a tendency in clinical practice to underestimate this persistent risk, emphasizing the need for increased awareness and vigilance in monitoring potential HCC development in this group [7,8].

Due to many medical conditions that can manifest fatigue, it is important to consider the coexistence of multiple diseases according to the patient’s other signs, co-morbidity, and risk factors. We present a challenging case of a 72-year-old man with a functional cure of hepatitis B, who had chronic fatigue and hyponatremia. Initially diagnosed with NFPA and having recovered from pituitary surgery, the patient later developed dyspepsia and recurrent fatigue, which led to a concurrent diagnosis of metastatic HCC.

## Case presentation

A 72-year-old male with a history of well-controlled hypertension, functional cure of hepatitis B, and stable coronary artery disease presented to the clinic with chronic fatigue for more than six months. In addition, the patient had hot flashes for more than one year and unintentional body weight loss of 14% reduction in three months, which occurred six months ago and did not decrease further. The physical examination showed bitemporal superior quadrantanopia, while both chest and abdomen examinations appeared normal. His volume status was deemed as euvolemic. A chest X-ray did not reveal any active pulmonary conditions.

Initial blood work (Table 1) was notable for anemia (hemoglobin 11.5 gm/dl), hyponatremia (sodium 127 mmol/dl), and low serum osmolality (osmolality 267 mosm/kgH_2_O). The spot urine analysis showed elevated urine osmolality and relatively low urine sodium. The hepatitis profile showed negative HBsAg and hepatitis C antibody (anti-HCV Ab) but positive hepatitis B surface antibodies (anti-HBs Ab) and hepatitis B core antibodies (anti-HBc Ab), confirming a functional cure of HBV infection. An endocrine evaluation (Table 2) revealed a low free thyroxine (T4) level (free T4 0.84 ng/dl) without an elevation in thyroid-stimulating hormone (TSH) level (TSH 2.84 μIU/ml), suggestive of secondary hypothyroidism. A low morning cortisol level (cortisol 4.47 mg/dl), coupled with decreased adrenocorticotropic hormone (ACTH) level (6.6 pg/ml) and an inadequate cortisol response to ACTH stimulation, revealed secondary adrenal insufficiency. Furthermore, the low androgen level (0.06 ng/ml) with normal luteinizing hormone (LH) and follicular-stimulating hormone (FSH) levels (LH 2.72 μIU/ml, FSH 6.4 μIU/ml) suggested hypogonadotropic hypogonadism. Collectively, these findings led to the diagnosis of panhypopituitarism.

**Table 1:** Initial laboratory data.

Parameters	Units	Values	Reference range
Serum			
Red blood cell count (RBC)	10^6^/μl	3.54	4.4 – 5.7
Hemoglobin (HGB)	g/dl	11.5	13.5 – 17.5
Mean corpuscular volume (MCV)	fl	90.7	85 – 101
Platelet count (Plt)	10^3^/μl	251	150 – 400
White blood cell count (WBC)	10^3^/μl	5.24	3.6 – 10.0
Creatinine	mg/dl	0.76	0.46 – 1.07
Sodium (Na)	mmol/l	127	135 – 148
Potassium (K)	mmol/l	3.9	3.7 – 5.3
Chloride (Cl)	mmol/l	90	96 – 110
Calcium (Ca)	mg/dl	8.6	8.6 – 10.2
Osmolality	mosm/kgH_2_O	267	275 – 294
Alanine aminotransferase (ALT)	U/l	10	4 – 44
Aspartate aminotransferase (AST)	U/l	24	8 – 38
Albumin	g/dl	4.4	3.8 – 5.3
Iron	mg/dl	73	30 – 170
Total iron binding capacity	mg/dl	294	200 – 400
Transferrin saturation	%	24.8	20 – 45
Ferritin	ng/ml	658	30 – 400
Spot urine			
Creatinine	mg/dl	154.8	
Sodium	mmol/l	20	
Osmolality	mosm/kgH_2_O	655	

**Table 2:** Endocrine function assessment.

Parameters	Units	Values	Reference range
Thyroxine (T4)	ng/dl	5.19	5.1 – 14.1
Free T4	ng/dl	0.84	0.93 – 1.7
Thyroid-stimulating hormone (TSH)	μIU/ml	2.84	0.27 – 4.20
Cortisol at 8 a.m.	μg/dl	4.47	6.02 – 18.4
Cortisol at 5 p.m.	μg/dl	1.99	2.68 – 10.5
Adrenocorticotropic hormone (ACTH)	pg/ml	6.6	7.9 – 47.1
Prolactin	ng/ml	12.1	4.0 – 15.2
Human growth hormone (HGH)	ng/ml	0.19	< 3
Follicular-stimulating hormone (FSH)	mIU/ml	6.4	1.5 – 12.4
Luteinizing hormone (LH)	mIU/ml	2.72	1.7 – 8.6
Testosterone	ng/ml	0.06	1.93 – 7.4
ACTH stimulation test (Tetracosactide 0.25mg intravenous injection)			
Cortisol in 30 minutes after injection	μg/dl	15.48	Cortisol < 18.1 μg/dl at 30 or 60 minutes indicate adrenal insufficiency.
Cortisol in 60 minutes after injection	μg/dl	17.19	

**Figure 1:**
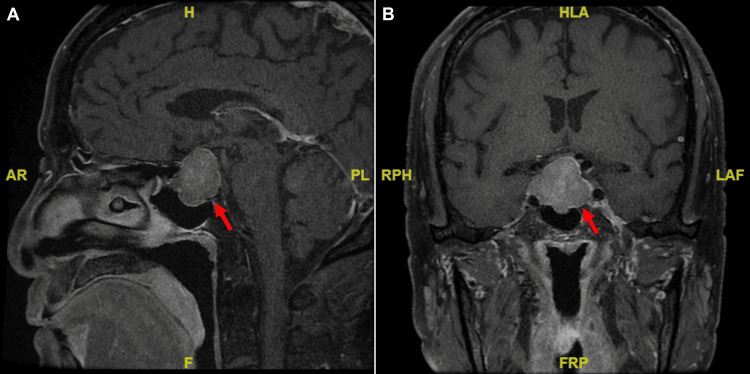
Contrast-enhanced longitudinal relaxation time (T1)-weighted magnetic resonance imaging depicts a pituitary macroadenoma (red arrows) measuring 3.1 x 2.8 x 2.2 cm, with evident compression of the optic chiasm observed in sagittal view (A) and coronal view (B).

**Figure 2:**
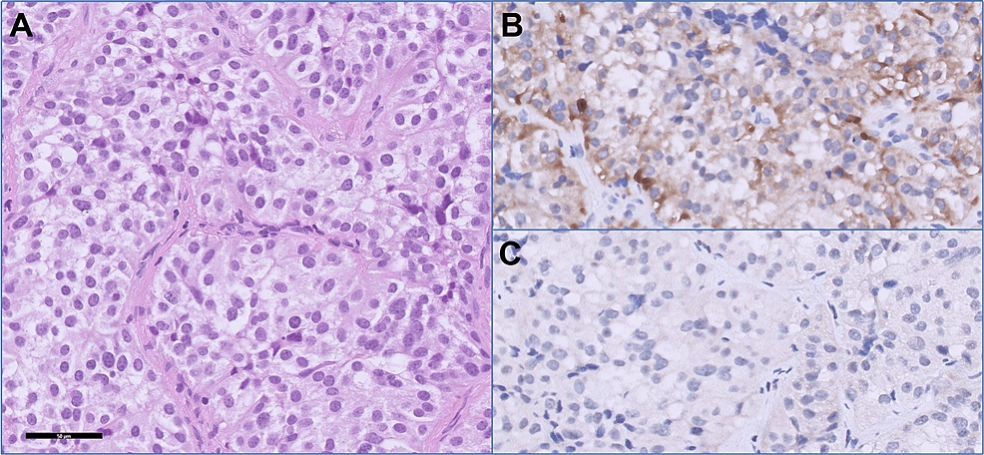
The hematoxylin and eosin stain of pituitary adenoma (A) reveals that the tumor is composed of round or polygonal cells with faintly eosinophilic to clear cytoplasm, arranged in sinusoidal and solid patterns. The immunohistochemistry results demonstrate positive staining for follicle-stimulating hormone (B) and negative staining for luteinizing hormone (C). Magnification: 400X.

Diagnostic imaging with pituitary magnetic resonance imaging (MRI) identified a pituitary macroadenoma (3.1 x 2.8 x 2.2 cm) with optic chiasm compression (Figure 1). Treatment with cortisone acetate 37.5 mg daily resulted in a notable improvement in fatigue and an increase in serum sodium levels from 127 mmol/l to 139 mmol/l within two weeks. A transsphenoidal resection of the pituitary macroadenoma was performed to address the symptoms of mass effect. Histopathological examination confirmed a gonadotroph adenoma, with FSH immunohistochemistry staining being positive (Figure 2). The postoperative follow-up revealed normalized free T4 and cortisol levels, while androgen levels remained low.

**Figure 3:**
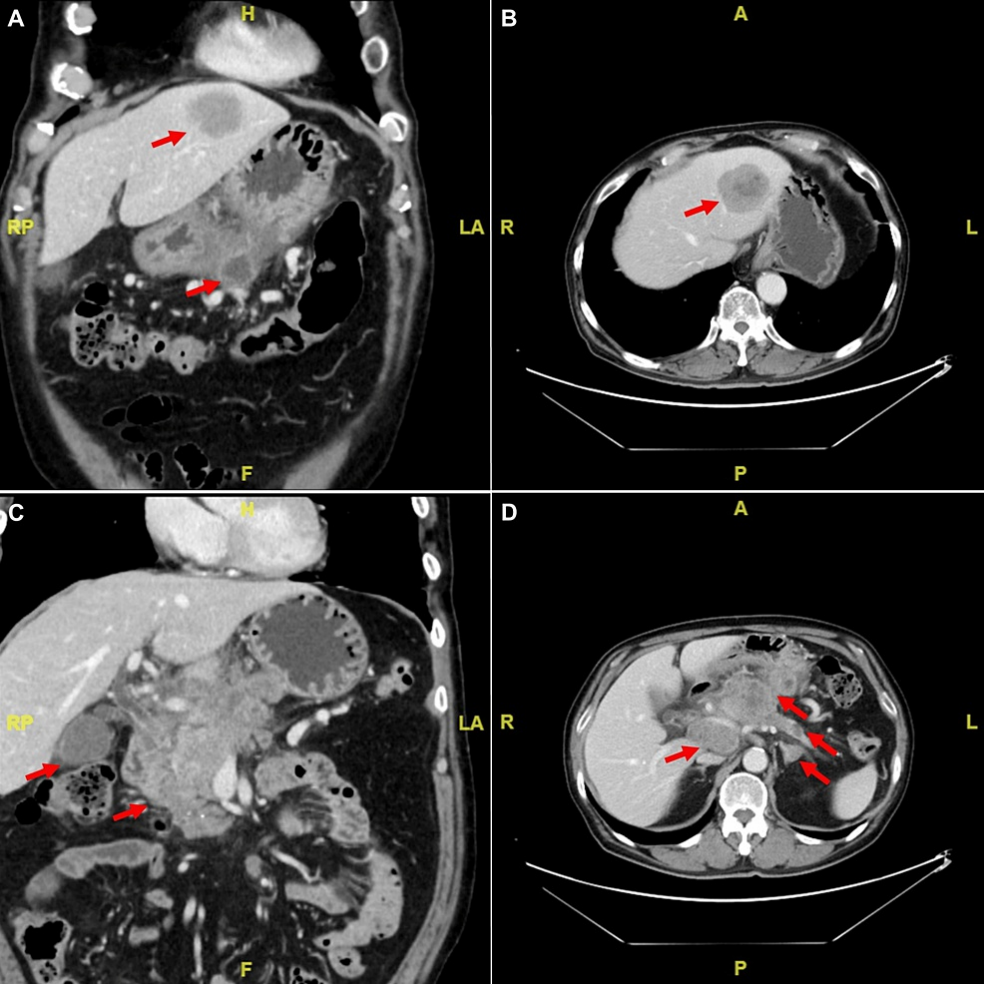
Contrast-enhanced computed tomography (CT) of the abdomen displays scattered tumors (red arrows) involving the liver and stomach in both coronal (A) and axial views (B). The CT also shows involvement of the pancreas, left adrenal gland, and peri-pancreatic lymph nodes in both coronal (C) and axial views (D).

**Figure 4:**
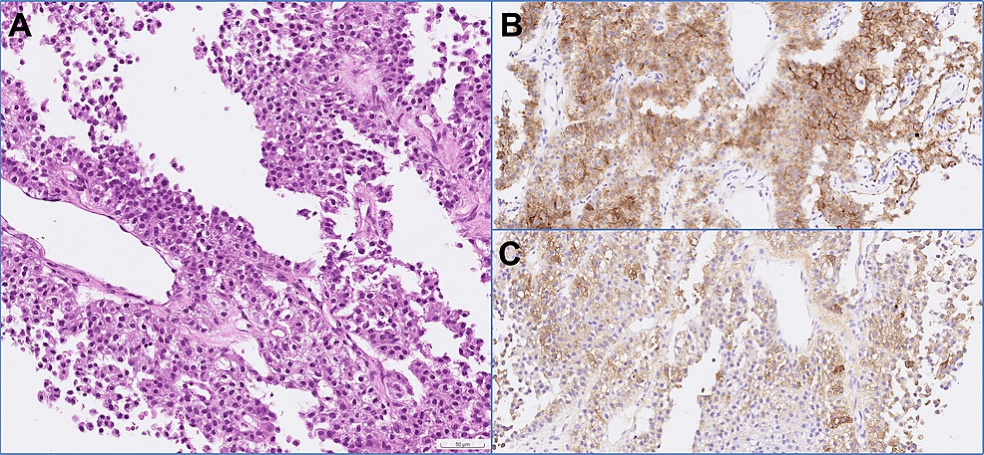
The hematoxylin and eosin stain of liver tumor biopsy (A) shows solid sheets of uniform tumor cells. The immunohistochemistry stain indicate diffuse immunoreaction with glypican-3 (B) and partial positivity for α-fetoprotein (C). These findings are compatible with hepatocellular carcinoma. Magnification: 200X.

Approximately two months after the surgery, the patient revisited the clinic, presenting with dyspepsia lasting for two weeks, along with easy satiety, hiccups, and recurrent fatigue. Abdominal examination did not reveal any tenderness or palpable masses. Bedside abdominal ultrasonography identified a hyperechoic, solid mass in the liver with an ill-defined border. Laboratory tests showed persistent anemia (hemoglobin 11.7 g/dl) and elevated α-fetal protein (AFP) (AFP 3387.9 ng/ml), but normal liver enzymes and undetected serum HBV DNA. Further diagnostic imaging with abdominal computed tomography disclosed scattered tumor lesions in the left lobe of the liver, pancreatic body, left adrenal gland, gastric body's posterior wall, and peri-pancreatic lymph nodes (Figure 3). Histological examination confirmed the diagnosis of HCC (Figure 4). Given the advanced nature of the disease, the patient was immediately referred to an oncology specialist for further evaluation and management.

## Discussion

In this report, we presented a 72-year-old man who experienced chronic fatigue and hyponatremia, leading to the dual diagnosis of NFPA and HCC. The suspicion of pituitary lesion was based on signs of bitemporal superior quadrantanopia and hyponatremia. The patient's hyponatremia was hypotonic and euvolemic, indicating normal body sodium with excess free water. A spot urine sample showed higher urine osmolality than plasma (Table 1), reflecting ADH activity on the collecting ducts causing water retention [12], even in the absence of dehydration. This clinical picture strongly suggested endocrine abnormalities, including adrenal insufficiency, hypothyroidism, or the syndrome of inappropriate secretion of antidiuretic hormone (SIADH). Additionally, the presence of normocytic anemia with normal levels of iron and total iron-binding capacity (Table 1) further supported potential endocrinopathies [13,14].

Endocrine function test (Table 2) diagnosed secondary adrenal insufficiency and hypothyroidism as contributors to the hypotonic hyponatremia. The former was confirmed by the ACTH stimulation test, showing a peak cortisol level of less than 18.1 mcg/dl at 60 minutes after cosyntropin administration [15]. The latter was inferred from the identification of a pituitary lesion and the absence of critical illness, excluding non-thyroid illness syndrome [16], despite not conducting a thyrotropin-releasing hormone (TRH) test. Hypogonadotropic hypogonadism was indicated by the patient's longstanding symptom of hot flashes and the pathology of the gonadotroph macroadenoma, despite not conducting an LH-releasing hormone (LHRH) test.

Interestingly, hyperprolactinemia is linked to 40% of the patients with NFPA due to pituitary stalk interruption of the dopaminergic axis [17]. The absence of hyperprolactinemia in our patient suggests that the dopaminergic axis was not significantly compressed. Moreover, although pituitary apoplexy could contribute to panhypopituitarism, the lack of ischemic or hemorrhagic features on the pituitary MRI [18] made this possibility unlikely.

Initially, abdominal imaging was deemed unnecessary due to apparent diagnostic clarity. It was only pursued when the patient developed abdominal symptoms and recurrent fatigue, leading to the discovery of metastatic HCC through a liver biopsy. This delayed diagnosis could have been avoided with earlier consideration of the patient’s risk for HCC, given his history of a functional cure of hepatitis B. Therefore, we recommend reassessing liver conditions in patients with a functional cure of hepatitis B who present with fatigue, to screen for potential HCC development.

The association between NFPA and an increased risk of malignancy remains controversial [19]; thus, the co-occurrence of NFPA and HCC in our patient may be coincidental. Intriguingly, the oncogene pituitary tumor-transforming gene 1 (PTTG1), first identified in pituitary tumors in rats [20], is expressed in nearly 90% of NFPA cases [21]. Additionally, PTTG1 is found in various cancer types, including HCC, where its high expression is associated with poor prognosis [22]. One study indicated that HBV could upregulate PTTG1, thereby promoting tumorigenesis in HCC [23]. Although it remains unverified whether PTTG1 is expressed in the pituitary and liver tumors of our patient, it is plausible that the concurrent presence of NFPA and HCC in this patient could be related to overexpression of the PTTG1 gene in both tumors.

Common metastatic sites of HCC include the lungs, adrenal glands, bones, and regional lymph nodes [24]. The extensive spread of tumors in our patient, particularly to the uncommon sites of the stomach and pancreas, raises the possibility of hepatoid adenocarcinoma originating in the stomach or pancreas. These malignant tumors share morphological similarities with HCC but are exceedingly rare [25]. Given that both AFP and glypican-3 can be expressed in these tumors [25], we cannot entirely rule out either diagnosis. Biopsies of the pancreas or stomach may be required for a differential diagnosis. However, even with biopsies from these sites, distinguishing between pancreatic or gastric hepatoid adenocarcinoma with liver metastasis and HCC with multiple metastases remains challenging. More critically, identifying the specific type of cancer offers minimal benefit to the patient since all three tumors carry a poor prognosis and lack effective treatment options.

Regardless of potential genetic correlations or specific tumor type, the unexpected subsequent diagnosis of HCC necessitates a reassessment of clinical reasoning processes. Clinicians often depend on the heuristic of Occam’s razor, which favors a singular, straightforward diagnosis to explain a patient’s symptoms [26]. While efficient, this approach risks missing multiple concurrent conditions with overlapping symptoms, which could delay the diagnosis of critical conditions, including malignancies. Therefore, clinicians should also consider Hickam's dictum, which advocates for a broader diagnostic lens, acknowledging the possibility of multiple co-existing conditions [27]. The value of Hickam's dictum is particularly pronounced in elderly patients, who are more likely to have multiple chronic illnesses and an accumulation of risk factors. To effectively address the challenges posed by complex cases, it is essential for clinicians to employ a balanced diagnostic approach. Integrating both Occam’s razor and Hickam's dictum enables a more nuanced understanding of a patient’s conditions, enhancing diagnostic accuracy and improving patient outcomes by ensuring timely and appropriate treatment strategies.

## Conclusions

This case highlights the necessity for a comprehensive and holistic diagnostic approach that encompasses the entire spectrum of potential diagnoses, even when initial clinical presentations may seem to point to a singular diagnosis. It also emphasizes the importance of maintaining a high level of suspicion for potential endocrine deficiencies in elderly patients presenting with symptoms such as fatigue and hyponatremia, as well as the persistent risk of HCC in patients who have achieved a functional cure of hepatitis B. By effectively balancing the principles of Occam's razor with Hickam's dictum, clinicians can improve diagnostic accuracy and enhance overall patient care.
